# Learning from machine learning: prediction of age-related athletic performance decline trajectories

**DOI:** 10.1007/s11357-021-00411-4

**Published:** 2021-07-09

**Authors:** Christoph Hoog Antink, Anne K. Braczynski, Bergita Ganse

**Affiliations:** 1 TU Darmstadt, Biomedical Engineering (KIS*MED), Darmstadt, Germany; 2 Department of Neurology, RWTH Aachen University Hospital, Aachen, Germany; 3 Institut für physikalische Biologie, Heinrich-Heine University Düsseldorf, Düsseldorf, Germany; 4 Innovative Implant Development, Department of Surgery, Saarland University, Homburg, Germany; 5 Department of Trauma, Hand and Reconstructive Surgery, Saarland University, Homburg, Germany

**Keywords:** Artificial intelligence, Track and field, Big data, Longevity, Ageing, Prediction

## Abstract

Factors that determine individual age-related decline rates in physical performance are poorly understood and prediction poses a challenge. Linear and quadratic regression models are usually applied, but often show high prediction errors for individual athletes. Machine learning approaches may deliver more accurate predictions and help to identify factors that determine performance decline rates. We hypothesized that it is possible to predict the performance development of a master athlete from a single measurement, that prediction by a machine learning approach is superior to prediction by the average decline curve or an individually shifted decline curve, and that athletes with a higher starting performance show a slower performance decline than those with a lower performance. The machine learning approach was implemented using a multilayer neuronal network. Results showed that performance prediction from a single measurement is possible and that the prediction by a machine learning approach was superior to the other models. The estimated performance decline rate was highest in athletes with a high starting performance and a low starting age, as well as in those with a low starting performance and high starting age, while the lowest decline rate was found for athletes with a high starting performance and a high starting age. Machine learning was superior and predicted trajectories with significantly lower prediction errors compared to conventional approaches. New insights into factors determining decline trajectories were identified by visualization of the model outputs. Machine learning models may be useful in revealing unknown factors that determine the age-related performance decline.

## Introduction

The inherent ageing process is associated with declines in physical performance that can partially be mitigated but currently not stopped or reversed [1, 2]. Frailty and sarcopenia, as well as chronic diseases, such as the metabolic syndrome, are often connected to a reduced quality of life in old age [3, 4]. Athletic performance declines in an almost linear fashion up until around the age of 70 years [5], when the decline progressively accelerates [6–10]. Physical performance decline trajectories vary among individuals, as reflected in longitudinal data [6, 11, 12]. People who participate in competitive sports longer were shown to experience a slower performance decline [13–15]. Further underlying factors for differences in individual decline trajectories are, however, poorly understood and their prediction thus poses a challenge. As an example, it is not clear whether athletes who perform better have a slower performance decline rate. In addition, the influences of diseases and injuries on the performance decline trajectories in various sports are unknown, despite the high relevance of this knowledge in an ageing society.

Large datasets and big-data approaches, such as computational models of performance decline trajectories, not only allow for predictions of future results, but may also help to identify factors associated with a particularly slow or fast decline [16, 17]. In the research of athletic performance declines, linear [5] and quadratic [10, 11, 15, 16] regression models are usually employed. However, these models suffer from the difficulty to consider individual factors and thereby often show high prediction errors when applied to the individual athlete. The main obstacle is to identify the relevant characteristics associated with a faster or slower decline rate. It has not yet been explored whether artificial intelligence applications, such as machine learning (ML) implementations, are potentially superior in delivering more accurate predictions.

Particularly, the prediction of the performance decline trajectory of an individual from only one measurement would be desirable, as it permits an immediate assessment without requiring results from several previous years that can often not be obtained. ML approaches were successful in the prediction of septic shock onset [18], epileptic seizures [19], the onset of type 2 diabetes mellitus [20], or ball trajectories for table-tennis robots [21]. In sports, the prediction of the potential and performance trajectories of young talents to identify future champions by ML has been demonstrated for archers [22] and in table tennis [23].

Track and field performance data are particularly suitable for computational modelling due to their highly standardized nature. The rules of competition [24] have been largely the same for more than a century [25], and results are assessed by objective measures, i.e. distances and times. Internationally, master athletics starts at the age of 35 years and athletes often continue to compete for several decades, leaving their trace of longitudinal performance data in the rankings databases. The largest longitudinal master athletics performance dataset published so far [11] is from the Swedish database “Swedish Veteran Athletics” [26] and covers 120 years, reaching back to 1901. The age range covered by the dataset is 35 to 97 years, and it includes 83,209 results from 34,132 male and female athletes. In the present study, we used a subset of these data to compare two straightforward decline prediction strategies with an ML approach comprised of a multilayer neuronal network. The aim was to compare their accuracy in predicting the performance decline trajectories for individual athletes from only one result.

We hypothesized that (1) it is possible to predict the future performance development of a master athlete from a single measurement, (2) prediction by an ML approach is superior to prediction by the average decline curve, (3) prediction by an ML approach is superior to prediction by an individually shifted decline curve, and that (4) athletes with a higher starting performance show a slower performance decline than athletes with a lower performance.

## Methods

The study was approved by the Institutional Review Board of the Faculty of Medicine of Rheinisch-Westfälische Technische Hochschule Aachen, Germany (reference number EK 300/17) and performed in accordance with the ethical standards of the 1964 Declaration of Helsinki and its later amendments.

### Data

From a Swedish longitudinal master athletics rankings dataset [11], the data of the following six sprinting and running disciplines were combined: 100 m, 200 m, 400 m, 800 m, 5 km, 10 km. Per definition, 100 m, 200 m, and 400 m are sprint disciplines; 800 m is a middle-distance running discipline; and 5 km and 10 km are long-distance running disciplines [27]. Due to a very low number of results from women in the dataset, only the results of men were included. These anonymized data include the best result a person has achieved at a given age for each discipline. The data are considered a time-unstructured dataset, which means available data points vary among subjects [28]. As the weights of the throwing devices (javelins, shots, hammers, and discusses) change stepwise with age, leading to an alteration of performance decline trajectories, throwing disciplines were excluded. Only data from athletes with n > 1 data points were selected. The first data point was used for prediction, and further data points of the individual were needed for model validation and comparison. Outliers were removed using thresholding and by applying Grubbs’s method [29]. The data of each discipline were normalized to the median value of the discipline at the age of 35 years by dividing the actual result at a given age by the corresponding median result at age 35 years, times 100, given in percent.

### Computational models

As a general assumption, a straightforward quadratic decline model was applied with *a*: age (years); P_35_: performance at age 35 (%); α_decline_: quadratic decline factor (%/years^2^); *i*: subject index.

1 $${\text{P}}^{i} _{{{\text{predicted}}}} \left( a \right) = {\text{P}}^{i} _{{{\text{35}}}} - \alpha ^{i} _{{{\text{decline}}}} (a^{i} - {\text{35}})^{{\text{2}}}$$

**Fig. 1:**
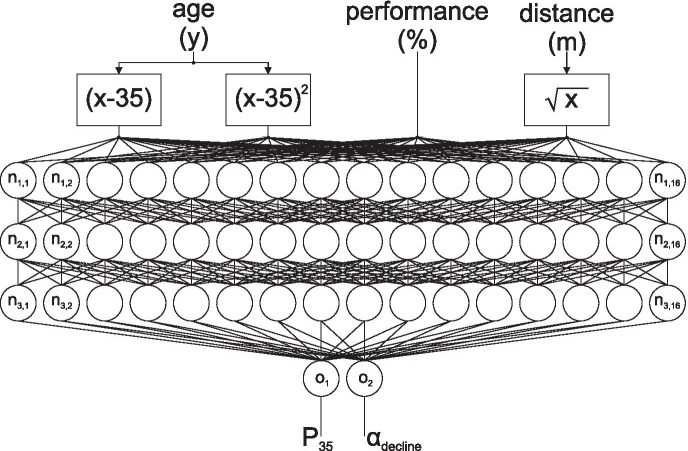
Depiction of the implemented neuronal network (ML prediction model). Three hidden layers were applied with 16 neurons each. Layers I and III used “exponential linear units” (ELUs) as activation functions; the middle layer II used “rectified linear units” (RELUs)

In the literature, typically pure quadratic or linear + quadratic declines are applied [5, 10, 11, 15, 16]. Just as in our previous work, we have chosen the more simplistic quadratic decline, as it is easy to interpret. According to this model, the predicted performance of a person at a certain age is a function of the person’s *estimated* starting performance at age 35 and the *estimated* decline factor. Thus, the task was to find both parameters, P^*i*^_35_ and α^*i*^_decline_, based on a single performance measurement of the respective individual *i* that allowed prediction of the performance trajectory with minimal error. Note that this implies that P^*i*^_35_ does not necessarily need to match the subject’s *actual* performance at the age of 35, P^*i*^_measured_(*a*^*i*^) with *a*^*i*^ = 35. We proposed three models for the determination of P^*i*^_35_ and α^*i*^_decline_:

1. “Global model”: P^*i*^_35_ and α^*i*^_decline_ were assumed to be constant for all athletes, P^*i*^_35_ = P_35, global_, α^*i*^_decline_ = α_decline, global_. To find both parameters, the following least-squares optimization problem was solved:
  $${\text{P}}_{{{\text{35}},{\text{ global}}}} ,\alpha _{{{\text{decline}},{\text{ global}}}} = {\text{arg min}}\left( {({\text{P}}^{i} _{{{\text{predicted}}}} \left( {a^{i} } \right) - {\text{P}}^{i} _{{{\text{measured}}}} \left( {a^{i} } \right)} \right)^{{\text{2}}}$$
  with P^*i*^_measured_(*a*) being the actual performance measurements of subject *i* at age *a*^*i*^, while P^*i*^_predicted_(*a*) is the corresponding prediction according to Eq. (1).
2. “Shifted global model”: α^*i*^_decline_ was constant for all athletes and determined in the same way as in the global model, α^*i*^_decline_ = α_decline, global_. P^*i*^_35_ = P^*i*^_35, shift_ was determined individually by shifting the decline curve to the actual starting performance P^*i*^_measured_(*a*^*i*^_start_) of the athlete *i* with the starting age *a*^*i*^_start_:
  $${\text{P}}^{i} _{{{\text{35}},{\text{ shift}}}} = {\text{P}}^{i} _{{{\text{measured}}}} \left( {a^{i} _{{{\text{start}}}} } \right) - \alpha _{{{\text{decline}},{\text{ global}}}} \cdot (a^{i} _{{{\text{start}}}} - {\text{35}})^{{\text{2}}}$$
  Note that this implies that in this scenario, P^*i*^_35_ matches the athlete’s actual performance at age 35 if that happens to be the first data point, i.e. *a*^*i*^_start_ = 35.
3. “ML prediction model”: Both, the starting performance and decline rate were computed individually by a multilayer neuronal network from the *i*th athlete’s starting age *a*^*i*^_start_, actual staring performance P^*i*^_measured_(*a*^*i*^_start_), and distance *d*^*i*^ in metres (Fig. 1):
  $${\text{P}}^{i} _{{{\text{35}}}} ,\alpha ^{i} _{{{\text{decline}}}} = {\text{NN}}\left( {{\text{P}}^{i} _{{{\text{measured}}}} \left( {a^{i} _{{{\text{start}}}} } \right),a^{i} _{{{\text{start}}}} ,d^{i} } \right)$$

All models assume a quadratic decline but differ in its estimation, as well as in the starting point.

The “ML prediction model” was implemented in PYTHON 3.6.9 using TensorFlow/Keras 2.4.1 and is visualized in Fig. 1: It consists of an input layer with four nodes, three hidden, fully connected layers (I, II, III) with 16 nodes each and an output layer with two nodes. The number of nodes was determined with an initial grid search. The four inputs to the model were the starting age of the athlete minus 35 (*a*^*i*^_start_ − 35), the square of the starting age of the athlete minus 35 ((*a*^*i*^_start_ − 35)^2^), the corresponding performance (P^*i*^_measured_(*a*^*i*^_start_)), and the square root of the distance of the discipline ($\sqrt{{d}^{i}}$). Note that in theory, the input of the squared starting age is not necessary, as this non-linear relationship could be approximated by an appropriate network. However, we chose this approach to facilitate learning by providing these inputs. The reasons are that the network is comparatively small, and since linear, as well as quadratic relationships of performance decline are usually reported in the literature [5, 10, 11, 15, 16]. Nevertheless, as by design of neural networks, the network has the capability to “ignore” our proposed inputs by optimizing the respective weights to zero. For the outer hidden layers (I and III), exponential linear units (ELUs) were used, while rectified linear units (RELUs) were used in the middle layer (II). All kernels were initialized with uniformly distributed random values. The code snippets to define the model and the loss function are given in the Appendix.

Compared to other big-data approaches, the amount of data was limited. Thus, 40-fold cross-validation was used for the evaluation of the “ML prediction model” as a compromise between runtime and fluctuation of results. In each step, 39/40 of the subjects were used for training, while the remaining 1/40 were used for prediction. After 40 steps, the data of all subjects were predicted while ensuring that no information could leak from the training to the test set. The heat maps presented in the manuscript were created by averaging the outputs of each of the 40 folds. For the evaluation of the “global model” and the “shifted global model,” leave-one-out cross-validation (i.e. 5439-fold cross-validation in this case) was used as the computational costs were extremely low and thus allowed this exhaustive evaluation.

Since the training of the network was initialized randomly and the amount of data for training was comparatively small, an ensemble approach was used to stabilize and improve results: instead of training one model, an ensemble of 20 models was trained with the same data but varying initializations. In the prediction stage, the output of all 20 models was averaged to generate the final prediction. Note that this implies that the presented heatmaps are averages of 20 models/fold × 40 folds = 800 model outputs.

### Prediction errors

For each model, the root-mean-square (RMS) prediction error was computed as follows. As the first result of an athlete was used to determine the parameters of the shifted global model and the ML prediction model, only the deviation of the predicted decline trajectory from the subsequent results was used to compute the errors. The RMS error is a measure of the differences between the actual and predicted values and therefore reflects the accuracy of the prediction. To determine differences in the error between models, significance was tested by the Wilcoxon rank-sum test by comparing the error values of all subjects for the three scenarios. This test was chosen since the data were not normally distributed, as indicated by the Kolmogorov–Smirnov test for normality.

## Results

**Fig. 2:**
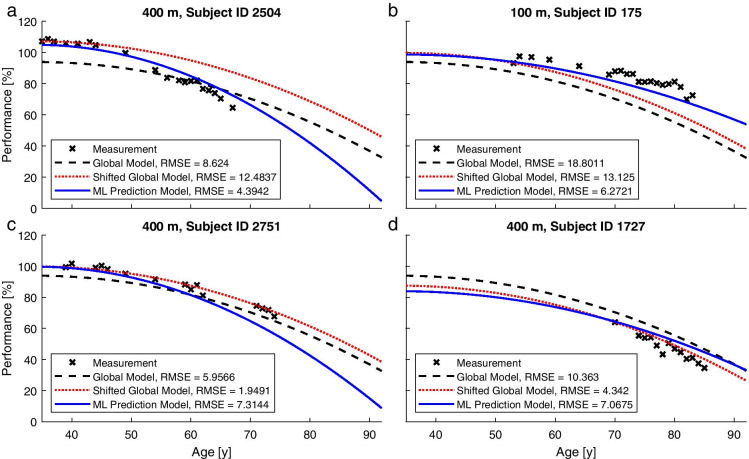
Examples of the actual data of four athletes and predicted performance declines of the three models. The examples were selected to showcase a variety of more extreme cases. The ML Prediction Model outperforms the other approaches in **a**) and **b**) but shows inferior performance in **c**) and **d**). RMSE = root-mean-square error

In total, 21,061 data points from 5439 male athletes were utilized. Further details on the data used for analysis are shown in Tables 1 and 2. Examples of the predicted performance trajectories of the three computational models are shown in Fig. 2.

**Table 1:** Details of the dataset

Discipline	Athletes	Data points	Mean data points per athlete	SD data points per athlete	Median data points per athlete	Maximum data points per athlete	Mean starting age (years)	Median starting age (years)	SD starting age (years)	Maximum starting age (years)	Mean span of data points (years)	Median span of data points (years)	SD span of data points (years)	Maximum span of data points (years)
100 m	560	2319	4.14	3.01	3	20	48.66	46	11.14	83	7.63	5	7.29	36
200 m	520	2165	4.16	2.97	3	19	49.25	46	11.33	81	7.36	5	7.30	39
400 m	557	2216	3.98	2.69	3	18	48.80	46	11.17	82	7.26	5	6.90	39
800 m	773	3062	3.96	2.65	3	18	48.15	46	10.69	82	7.52	5	7.41	42
5 km	1505	5723	3.80	2.61	3	19	46.49	44	9.94	79	6.14	4	6.11	42
10 km	1524	5576	3.66	2.40	3	18	45.11	43	9.32	76	5.83	4	5.97	43
Combined	5439	21,061	3.87	2.65	3	20	47.07	45	10.40	83	6.64	4	6.64	43

**Table 2:** Numbers of results per age group and discipline

Discipline	Age group											
	35–39	40–44	45–49	50–54	55–59	60–64	65–69	70–74	75–79	80–84	85–89	90–94
100 m	241	401	325	271	234	222	218	200	128	54	20	5
200 m	221	358	293	275	214	191	223	208	112	49	16	5
400 m	255	339	315	279	225	231	223	196	85	47	17	4
800 m	382	475	436	387	324	364	286	219	111	60	18	0
5 km	931	1030	927	776	583	536	442	295	147	49	7	0
10 km	1159	1114	942	748	521	436	358	213	73	11	1	0
Combined	3189	3717	3238	2736	2101	1980	1750	1331	656	270	79	14

### Prediction errors

**Fig. 3:**
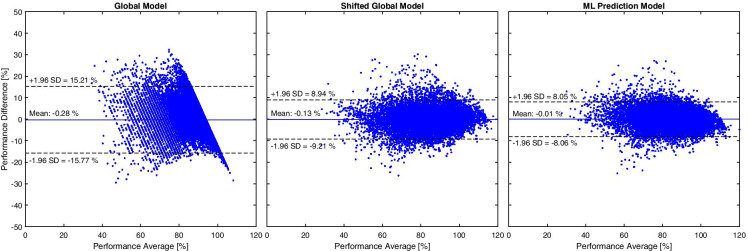
Bland–Altman plots showing the prediction errors of the three models. Each data point marks the best result of one subject in 1 year, i.e. multiple data points per subject exist. The systematic bias (mean) and the limits of agreement (± 1.96 standard deviations) are decreasing from global model over shifted global model to ML prediction model, indicating an increase in performance

**Fig. 4:**
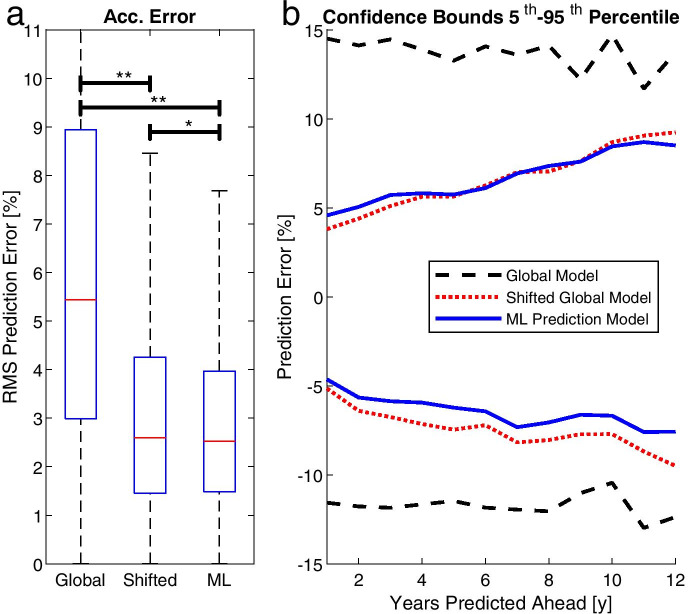
Error statistics of the three models. **a** Root-mean-square prediction error. * *P* < 0.05, ** *P* < 0.001, determined by rank-sum test. The error was accumulated, i.e. one data point exists per subject, even if the athlete has competed multiple times (see also Fig. 2). **b** Prediction errors shown in confidence bounds. If, for example, the performance of an athlete is predicted 6 years into the future using the shifted global model (the ML prediction model), 5% of predictions have an error greater than 6% (6%), and 5% of the data have an error smaller than − 7% (− 6%)

Figure 3 shows the prediction errors of the three models as computed by RMS prediction. The RMS error was significantly lower in the ML model compared to the other models (Fig. 4). The global model had by far the highest error. The difference was highly significant compared to both, the shifted global and ML models. The shifted global model was again associated with a significantly higher error than the ML model. Figure 4b shows the prediction error confidence bounds plotted for the years predicted ahead for all subjects. We defined the confidence bounds as the 5th and 95th percentile. The figure indicates that for the shifted global model and the ML model, the confidence bounds of the prediction error started at approximately ± 5% for predictions 1 year into the future and increased linearly with a growing number of predicted years. This was not the case for the global model. However, its confidence bounds exceeded those of the other models and were approximately constant at + 15 ± 12%, while the shifted global model and the ML model exhibited a confidence bound of approximately ± 8% for predictions 10 years into the future. Additionally, the lower bound of the ML model was approximately one percentage point better than those of the shifted global model. Although statistically significant, the differences between the shifted global model and the ML model were small in absolute numbers, namely 0.77 percentage points in terms of the upper adjacent and 0.07 percentage points in terms of the median (Fig. 4a).

### Factors determining performance decline trajectories

**Fig. 5:**
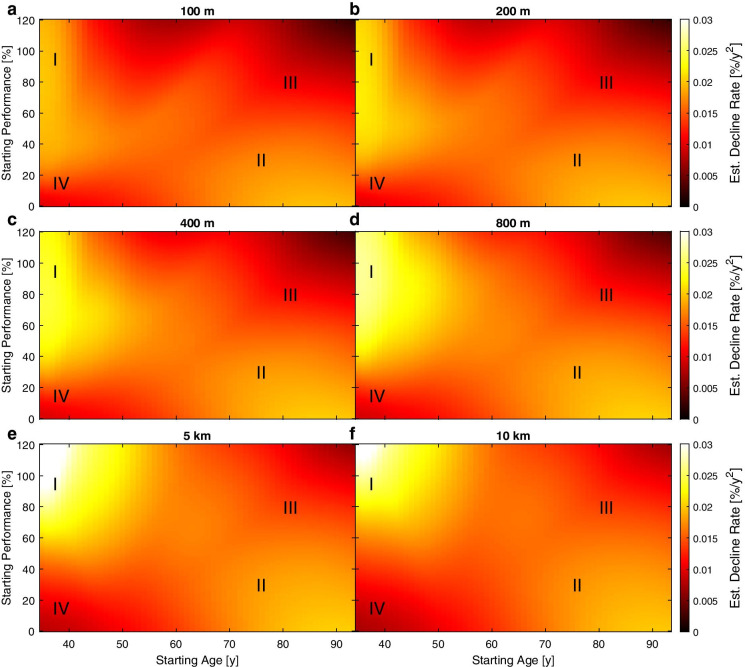
Heat maps for all disciplines with increasing distance from **a**) to **f**), showing the estimated decline rate in colour-coding, as calculated by the ML model. The roman numerals indicate areas of interest: I starting young with high performance, II starting old with low performance, III starting old with high performance, and IV starting young with very low performance

To answer the question if better performers have a slower performance decline than athletes who performed worse, data were visualized by heat maps that depict the outputs of the ML model for each of the six disciplines analyzed. The estimated performance decline rate (Fig. 5) was highest in athletes with a *high* starting performance and a *low* starting age (marked “I” in Fig. 5), as can best be seen in 10-km runners (Fig. 5b). A second group of athletes with a higher-than-average performance decline rate was found to have a *very low* starting performance and *high* starting age (marked “II” in Fig. 5). The lowest decline rates, on the other hand, were found for athletes with a *high* starting performance and a *high* starting age (marked “III” in Fig. 5). In particular in the middle- and long-distance runs, a very low decline rate was also found for athletes with a *very low* starting performance and *low* starting age (marked “IV” in Fig. 5).

Differences between disciplines were variations in the intensity of these findings, as shown in Fig. 5. All decline rates tended to be higher for 5 km compared to 100 m, as is evident from the different overall level of brightness in the heat map. The general picture, however, showed up in all disciplines: The highest performance decline rate was observed in athletes with a *high* starting performance and *low* starting age (marked “I” in Fig. 5) and was especially prominent in the long-distance (5 km and 10 km) runs. The lowest performance decline rate was found in the sprints and 800 m in athletes with a *high* starting performance and *high* (marked “III” in Fig. 5) starting age.

If we split the data of all disciplines into four quadrants with the thresholds *a*_start,th_ = 60 a, P_measured_(*a*_start_) = 60% (i.e. group I: *a*_start,th_ < 60 a, P_measured_(*a*_start_) > 60%, group II: *a*_start,th_ > 60 a, P_measured_(*a*_start_) < 60%, …), the number of subjects per group are I = 4700, II = 55, III = 579, IV = 2. In addition, as shown in our previous work, there are individual “outliers” in group I that have an impressive number of measurements, i.e. athletes who have competed for more than 16 (not necessarily consecutive) years. In terms of estimation error of the ML model, however, we did not detect striking differences between these groups.

### Starting performance

**Fig. 6:**
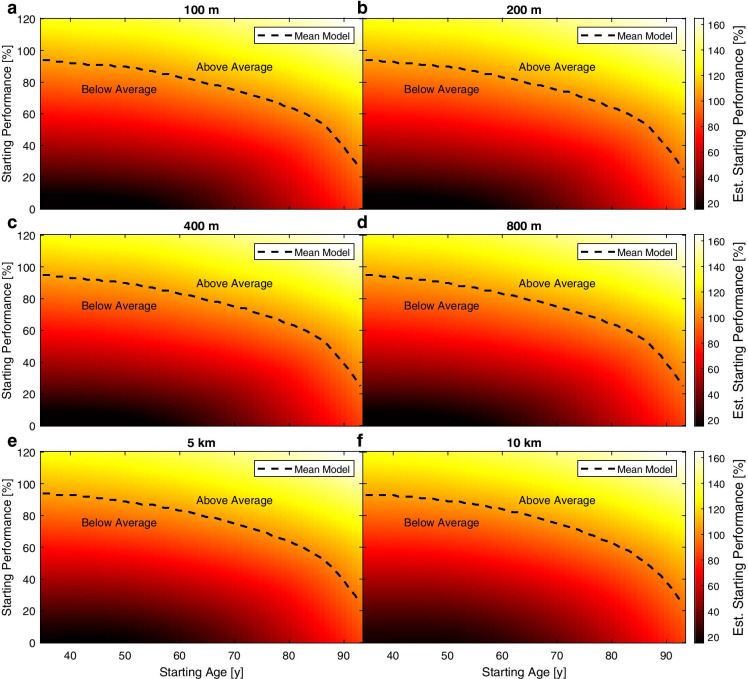
Heat maps for all disciplines with increasing distance from **a**) to **f**). The estimated starting performance (as calculated by the ML model) is colour-coded. It was highest in athletes with a high starting performance and a high starting age. The mean is indicated by a dashed line. Note that the relationship is not linear and shows a bend at around 85 years in all disciplines towards a higher starting performance

In Fig. 6, the estimated starting performance of the ML model is colour-coded for each discipline. It was highest in athletes who had a *high* starting performance and a *high* starting age, and lowest in those with a *low* starting performance and *low* starting age. The relation was not linear, as one might expect, but followed a more quadratic behaviour. Moreover, a sudden increase in the tendency towards a higher starting performance was found around the age of 85 years and older in all disciplines. This drop cannot be explained by lower numbers of athletes in these age groups (Table 2).

## Discussion

In the present study, we showed that (1) it is possible to predict the future performance development of a master athlete from a single measurement, and that (2) the prediction by an ML approach is superior to the prediction by a naïve average approach, and (3) to the application of a constant decline rate with individualized starting points. Interestingly, (4) the estimated performance decline rate was highest in athletes with a high starting performance and a low starting age, as well as in those with a low starting performance and high starting age, while the lowest decline rate was found for athletes with a high starting performance and a high starting age. This tendency was the same for all disciplines, while the absolute values of the decline rate varied.

Performance prediction from a single value is of interest for clinical practice, frailty research, athletes, and insurance companies. The ML model presented in this paper is potentially applicable to other scenarios in ageing, given an appropriate dataset is available, such as declines in hand grip strength [30], other measures of sarcopenia and frailty [31, 32], or bone density and fracture risk prediction [33, 34].

The present study tested a machine learning approach and showed its superiority to traditional approaches in the prediction of age-related master athletics performance decline trajectories. These differences, however, were small in absolute values. This comes as no surprise, given the seemingly impossible nature of the task of predicting performance years in advance from a single measurement without any additional information, such as the individual health status or training habits. Nevertheless, the differences in prediction accuracy proved to be statistically significant. Far more important, however, is the fact that we visualized the output of the ML model, thereby revealing the learned non-linear mapping between the three inputs “discipline”, “starting age”, and “starting performance” and the output of the predicted parameters of a performance decline curve. We believe that our approach showcases the possibility to learn from machine learning, i.e. using (at least seemingly) black-box systems to reveal aspects that may not be detected otherwise and that may be worth additional scientific attention and analyses.

The ML model delivered new insights into factors that determine performance decline trajectories in master athletes. Our findings on factors that influence a slower or faster performance decline and the identified criteria are entirely new and have to our knowledge not been previously reported. It has been speculated that regular physical activity is associated with a better general fitness [35] and it was suggested to flatten the physical performance decline trajectory, also with regard to the VO2max decline [13–15]. Our findings confirm this theory, as associations of the starting age and starting performance with the performance decline rate were identified. The estimated performance decline rate was highest in athletes with a high starting performance and a low starting age, as well as in those with a low starting performance and a high starting age. The phenomenon that a high starting performance between 35 and 40 years is connected with a high decline rate could potentially be explained by reductions in training volumes. Individuals in this age group often have less free time than before due to their family and career, potentially coming from very high training volumes in their 20 s and early 30 s. Injuries and degeneration may contribute to the performance decline [36]. Since the reported performance decline rate in this age group is usually very low [5, 10, 11, 15, 16], these findings were surprising and should be followed up in future research. A high decline rate in athletes with a low starting performance and a high starting age is in line with the theory that lifelong exercise helps to flatten the performance decline curve that would in turn be steeper in those who have not exercised continuously [13–15]. In addition, we interpret the output of our model as an indication that individuals who start late can still achieve a lower performance decline rate. This is in line with the finding that master athletes maintain better health than age-matched non-athletes [35]. We also found low decline rates for individuals with a very low starting performance and low starting age. However, these results need to be interpreted with extreme caution, because although seemingly reasonable (“whoever starts low can only decline so much”), there were virtually no data points in that area to allow for the model to properly learn about this group.

The lowest decline rate was found for athletes with a high starting performance and high starting age. A high performance at a high starting age may result from an ongoing, lifelong engagement in other sports and physical activities or from a combination of factors including nutrition and a good genetic constitution. Unfortunately, we do not have data on the amount of exercise and other biographic, genetic, or socioeconomic aspects of these athletes, and we can therefore only speculate on the underlying causes.

Our work has some limitations: First, the number of nodes in the model (n = 16) was determined via an initial grid search on the data. This can be regarded as information leakage and might be associated with overfitting. However, we re-evaluated the presented analysis with various values of n (n = 10, n = 12, n = 14, n = 18). In this analysis, we found that the model behaves essentially the same for all tested values of n. For example, all Bland–Altman plots (Fig. 3) showed an absolute value of the mean smaller than 0.06%, an upper bound smaller than 8.14% and a lower bound greater than − 8.13%. The difference between the ML model and the shifted model was always significant (p < 0.05, Fig. 4). Moreover, the four regions identified in Fig. 5 were visible for all variations of n. We thus conclude that the model is fairly insensitive to the exact number of nodes n. Nevertheless, the ML model was not validated against a completely independent hold-out test set due to the limited amount of data, but a cross-validation regime was used. We chose this approach, as the main focus of the work was on the interpretation of the model output and not to find the best possible or most robust ML model. In addition, we plan to test the model on additional independent datasets once they become available.

Note that cross-validation ensures a complete separation of the training and test set, and thus minimizes the risk of overfitting. Still, the ML model has far more parameters than the global model or the shifted global model and we can therefore only speculate about its performance on data recorded under vastly different conditions, which remains to be evaluated. Thus, if only the prediction of the performance trajectory is of interested, the shifted global model might be the preferred option. If the focus, however, lies on the extraction of novel information (e.g. the identification of subgroups in this work), the ML model is more suitable.

The dataset that we have used here only contains the best performance of a person for each age this person has competed in, but no further information. We hope to obtain longitudinal datasets with additional information, such as training volumes and diseases in the future. Finally, due to the low number of women in the dataset, only men were analyzed, and it remains unknown if the same findings apply to women [37]. Although this is a problem common to many medical-related AI studies, we acknowledge the severity of the implications and plan to follow up on this issue once more data will be available.

In conclusion, for the prediction of performance decline trajectories of master athletes based on one measurement, an ML approach in terms of a multilayer neuronal network showed lower prediction errors and was thereby superior to traditional approaches. ML models should be explored further in big-data research on age-related performance decline rates, in particular in two ways: First, to optimize prediction results, the possibility to integrate more data (i.e. more measurement points, additional individual information, external factors, etc.) should be studied. Second, the potential of ML models to identify relevant factors can be explored in other ageing-research areas or with additional model inputs.

## Data Availability

All data are available on the website of the Swedish Master Athletics Data Base [26] that was scraped as described in [11]. In addition, we are happy to share the data upon reasonable request.

## Appendix ML model code snippet

The customized loss function “my_loss_fn” was implemented to minimize the root-mean-square difference of measurements and predictions based on the outputs of the neural network. In it, “y_pred” was the model output in the form of a column vector. The first column contained the values for P^*i*^_35_. Note the scaling with 140 in line 6 to allow the model output to lie between 0 and 1. The second column contained the values for α^*i*^_decline_. The column vector “y_true” contained the measured data to be predicted. Here, the first column contained the pre-calculated quantities “age minus 35 squared” (*a*^*i,j*^-35)^2^, while the second column contained the corresponding desired output P^*i,j*^_measured_(*a,*^*i,j*^), where *i* was the index of the subject and *j* was the index of the data point of that subject. Note that *j* > 1 as *a,*^*i,j*=1^ = *a*^*i*^_start_.

The “adam” solver with standard parameters (learning rate = 0.001, β_1_ = 0.9, β_2_ = 0.999, ε = 1e-07) was used for optimization. Moreover, the batch size was set to 300 and the number of epochs to 200.

## Code availability

The essential part of the code is given in the methods section. Additional code is available upon request from the authors.
